# A CNN Deep Local and Global ASD Classification Approach with Continuous Wavelet Transform Using Task-Based FMRI

**DOI:** 10.3390/s21175822

**Published:** 2021-08-29

**Authors:** Reem Haweel, Noha Seada, Said Ghoniemy, Norah Saleh Alghamdi, Ayman El-Baz

**Affiliations:** 1 Faculty of Computer and Information Sciences, University of Ain Shams, Cairo 11566, Egypt; reem.t.haweel@cis.asu.edu.eg (R.H.); noha.seada@cis.asu.edu.eg (N.S.); said.Ghoniemy@cis.asu.edu.eg (S.G.); 2 Bioengineering Department, University of Louisville, Louisville, KY 40208, USA; 3 College of Computer and Information Science, Princess Nourah Bint Abdulrahman University, Riyadh 11671, Saudi Arabia; nosalghamdi@pnu.edu.sa

**Keywords:** autism, ASD, computer-aided diagnosis, deep learning, CNN, CWT

## Abstract

Autism spectrum disorder (ASD) is a neurodegenerative disorder characterized by lingual and social disabilities. The autism diagnostic observation schedule is the current gold standard for ASD diagnosis. Developing objective computer aided technologies for ASD diagnosis with the utilization of brain imaging modalities and machine learning is one of main tracks in current studies to understand autism. Task-based fMRI demonstrates the functional activation in the brain by measuring blood oxygen level-dependent (BOLD) variations in response to certain tasks. It is believed to hold discriminant features for autism. A novel computer aided diagnosis (CAD) framework is proposed to classify 50 ASD and 50 typically developed toddlers with the adoption of CNN deep networks. The CAD system includes both local and global diagnosis in a response to speech task. Spatial dimensionality reduction with region of interest selection and clustering has been utilized. In addition, the proposed framework performs discriminant feature extraction with continuous wavelet transform. Local diagnosis on cingulate gyri, superior temporal gyrus, primary auditory cortex and angular gyrus achieves accuracies ranging between 71% and 80% with a four-fold cross validation technique. The fused global diagnosis achieves an accuracy of 86% with 82% sensitivity, 92% specificity. A brain map indicating ASD severity level for each brain area is created, which contributes to personalized diagnosis and treatment plans.

## 1. Introduction

Autism spectrum disorder (ASD) is a neurodevelopmental disorder that affects social communication ability. ASD also causes language impairment and repetitive behaviors [1]. Individuals with ASD show different severity levels associated with each symptom [2]. The common ASD diagnostic standard utilizes history and expert clinical judgment together with behavioral modules of the autism diagnostic observation schedule (ADOS) [3,4]. Autism is diagnosed with the arising noticeable symptoms which start at the age of three to five years [5]. It is crucial to intervene and diagnose ASD early to allow for better assessment and treatment.

ASD can be diagnosed at the age of 12 months old, especially with the emergence of imaging diagnostic tools that employ brain imaging modalities such as structural (sMRI), functional (fMRI), and diffusion (DTI) magnetic resonance imaging [6]. Combining these scans to view the structure of the brain together with the brain functional activity during rest and performance of certain tasks constitute an early biomarker for ASD [7].

Resting state and task-based fMRI are types of fMRI scans that are adopted to manifest functional activity. Task-based fMRI measures evoked blood oxygen level-dependent (BOLD) signals during the performance of different tasks [8] such as auditory tasks, language tasks, visual processing tasks, motor tasks, and social tasks [9].

To investigate autistic brain abnormal functional response to speech compared to typically developed (TD) peers, several studies were performed [10]. Studies in [11,12,13] played an audio of a simple bedtime story and examined the sleep fMRI response. These studies included 40 autistic toddlers and 40 TD toddlers with ages that range from 12 to 48 months. Autistic toddlers showed abnormal laterality and hypoactivation in the left anterior portion of the superior temporal cortex (aSTG). On the other hand, TD toddlers exhibited the normal dominant activation of the left hemisphere aSTG. They also suggested early intervention and treatment as they demonstrated that as the age increases, lateralization abnormality increases.

Several studies up to 2013 that were reviewed in [14] concluded the involvement of atypical lateralization with language impairment. Individuals with ASD exhibited attenuation in the left hemisphere activation. Also, anomalous lateralization in the functional areas responsible for prelinguistics and language, specifically the fronto-temporal regions, were present. One of the reviewed studies [15] revealed atypical lateralization starts at an early age. Lower lateralization was present in high risk ASD infants, while higher lateralization was present in low risk peers. A review in [16] concluded similar results.

A meta-analysis of fMRI studies until 2013 was presented in [10]. Increased activation in the right precentral gyrus and decreased left activation were revealed in ASD individuals who performed language and auditory tasks, which contradicts the normal activation in TD individuals. Moreover, fMRI scans in TD individuals showed higher activation in the bilateral superior temporal gyri (STG) and left cingulate gyrus than ASD peers.

Literature on task-based fMRI analysis for ASD concludes fundamental differences in activation in ASD compared to TD individuals. These findings support the employment of task-based fMRI for early ASD diagnosis [17]. Machine learning (ML) has made it possible to develop intelligent and automated systems for several pattern recognition applications. The emergence of noninvasive or minimally invasive medical screening devices created massive informative data structures that allowed for the exploitation of ML for automated diagnosis. A research in [18] proposed a pipeline based on task fMRI scans for predicting treatment of social responsiveness scale outcome. They applied the general linear model (GLM) for brain feature extraction. Feature selection techniques were performed following feature extraction. For classification, they employed the random forest (RF) classifier. Twenty ASD children (5.90 ± 1.07 years) were included in the study. A recent study in [19] performed both local and global diagnosis for ASD toddlers. Brain areas parcellated with the Brainnetome atlas (BNT) were analyzed with a stacked nonnegativity constraint auto-encoder. The study included 30 ASD against 30 TD and classified between two groups with an accuracy of 75.8%. Another recent study graded the severity of autism into three groups [20,21]. GLM analysis for low individual level analysis, to extract features, and high group level analysis, to infer statistical differences between groups and validation, were applied. They utilized different approaches to extract features from GLM analyzed whole brain areas. Among the several classifier architectures they tested, Random Forest performed best with 78% accuracy. In [22], they enhanced their framework by performing a two stage classifier, included more data (92 mild, 32 moderate, and 33 severely autistic) and performed more validation techniques. Accuracies ranged between 70% and 83%.

ML and deep learning, which is a subset of ML that involves deep networks, have played a very important rule in many neuroscience applications. Convolutional neural network (CNN) is one of the most powerful DL network architectures. CNNs are deeply adopted in Brain-Computer Interfaces (BCI) as well as classification of EEG signals [23,24,25].

Recently, CNNs have been widely utilized for ASD diagnosis and analysis with fMRI [26]. Jinlong Hu et al. [27] adopted a multi-channel 2D CNN model to classify FMRI dataset of 995 subjects in a motor experiment. They proved that CNNs achieve good performance with high dimensional data, in comparison with other classifiers, mostly when the dataset is large as in their case. A study in [28] investigated the employment of spatial and temporal features of task-based fMRI. To capture the spatial information, they developed a 3D convolutional neural networks on two-channel images of mean and standard deviation that were created by the sliding window, which captures the temporal statistics. This framework achieved an 8.5% increase in the mean F-scores.

FMRI scans constitute 4D data of a brain 3D volume consisting of 1D time-dependent BOLD signals. Several signal processing techniques can be optimized to analyze these BOLD signals. Wavelet transform are considered one of the efficient time signal processing techniques for resolving time-series. Applications of the wavelet transform include compression, high resolution time, and frequency analysis and denoising [29]. It has also been utilized for fMRI analysis as an alternative to conventional GLMs. PS Lessa et al. [30] concluded that Wavelet correlation analysis achieves higher statistical power in comparison to GLMs. Moreover, wavelet transforms contribute to the achievement of efficient brain disorder diagnosis, such as ADHD, autism and Alzheimer diagnosis, when applied on fMRI feature processing. In an approach to diagnose ADHD, García et al. [31] performed continuous wavelet transform (CWT) to create scalograms of BOLD signals.

Most previous fMRI experiments were applied on adults [32,33], however, our proposed study includes toddlers/infants from 12 to 40 months old. The aim of our study is to develop an early autism computer aided local and global detection tool. Spatial dimensionality reduction with region of interest (ROI) selection and clustering have been performed to reduce the 4D fMRI data to a reduced number of BOLD signals. In order to provide a detailed frequency and scale representation, we have applied CWT on selected BOLD signals. CWT creates scalogram images that are used as input images to multi-channel 2D-CNNs for each area. Finally, brain maps that indicate level of ASD severity for each ROI is provided for each subject. The proposed framework works towards determining the neuro-circuits with abnormalities as well as creating personalized diagnosis and treatment plans that handles the specific case of each individual. Moreover, CWT achieved better results compared to other feature extraction and generation techniques.

## 2. Materials

### 2.1. fMRI Data Collection

This study includes subjects from “Biomarkers of Autism at 12 Months: From Brain Overgrowth to Genes” dataset. This dataset was collected between August 2007 and June 2014 and is provided by the national database for autism research (NDAR: http://ndar.nih.gov (accessed on 22 May 2019)) [11,34,35]. The dataset included 639 subjects that were tracked every 12 months roughly starting at 12 months and until they are 40 months old.

We have chosen some substantial criteria in selecting subjects for our study such that included subjects must have ADOS toddler module, sMRI (T1) and (T2), and response to speech task fMRI (T2*). Intensive validation on each report and scan has been conducted. Visual validation is performed for all sMRI scans to exclude inaccurate or corrupted ones. FMRI scans have been validated to have 154 volumes and visually validated to have no clear artifacts. One hundred subjects (50 ASD 50 TD) with ages ranging between 12–40 months old, are included in this study. Information about each subject, such as IDs and final diagnosis, as well as the extracted BOLD signals of this dataset are available in Supplementary Materials S1 and S2, respectively.

### 2.2. Response to Speech Experiment

The experiment that was used while task-based fMRI scans were acquired is a response to speech experiment. An audio record of a narrator telling a story was played during natural sleep. The audio consists of three different types of records, simple forward speech, complex forward speech, and backward speech. Such records alternate with silence periods and are repeated during a 6 min and 20 s span.

## 3. Methods

**Figure 1:**
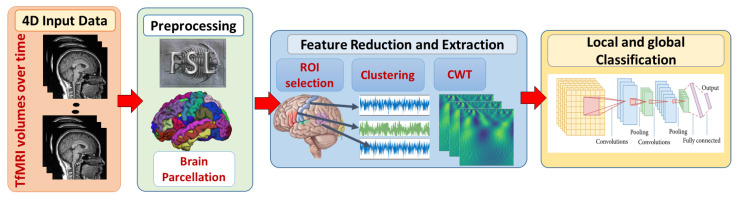
The proposed framework for local and global classification. First, 4D fMRI data are preprocessed with FSL. Brain extraction and parcellation to Harvard-Oxford probabilistic atlas are also performed. Second, spacial and temporal feature reduction and extraction techniques are performed. Finally, local classification models on each ROI are developed to provide a global classification decision.

In this study, local and global ASD diagnosis have been developed. Figure 1 demonstrates the adopted framework. First, fMRI scans are preprocessed using FMRI expert analysis tool (FEAT) [36] developed in fMRI’s software library (FSL) [37]. Brain parcellation is based on Harvard-Oxford probabilistic atlas https://identifiers.org/neurovault.collection:262. (accessed on 11 April 2019) The Detailed explanation of preprocessing steps is provided in [20].

### 3.1. Spatial Dimensionality Reduction

Applying neural networks on raw data without feature engineering is feasible when the raw data are easily separable. However, identifying autism biomarkers in task fMRI is a complex problem as autism follows a wide spectrum and is not easily separable. Moreover, fMRI raw data is a high dimensional data of 4D. CNN performance decreases when data dimensionality is high and input data size is small as in medical applications. Hence, it is crucial to reduce dimensionality. A comparison of fMRI feature extraction and reduction approaches have been presented in [38], proving higher ASD classification results. The following steps have been proposed for feature reduction:

- ROI selection: Based on literature of the response to speech experiment for toddlers, specific brain areas related to language circuits are activated. These areas include cingulate gyri (CG), superior temporal gyrus (STG), primary auditory cortex (PAC) and angular gyrus (AG) for both hemispheres. In this study, the most significantly activated brain areas are selected.
- Clustering: Each brain includes several commonly activated voxels, which are considered redundant data. Therefore, grouping similar BOLD signals in each area and extracting a single value for each group is efficient and can extensively enhance classification performance. Hence, each brain area’s BOLD signals have been clustered with kmeans. Different number of clusters have been tested to achieve higher validation accuracies.Two methods to represent the signals of each cluster have been tested: averaging BOLD signals, or extracting the BOLD signal closest to the center of that cluster.

The advantage of the previous reduction approaches is that the brain structure is maintained. Each brain area is represented by a number of features. This technique allows for local analysis and obtaining brain maps.

### 3.2. Continuous Wavelet Transform

**Figure 2:**
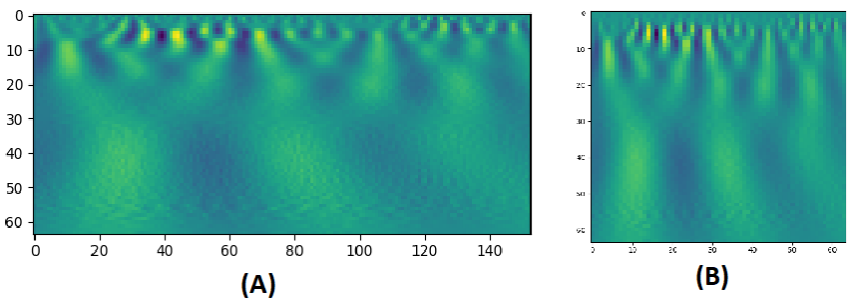
(**A**) A CWT scalogram example with 64 scales of a BOLD signal of 153 time points. (**B**) The resized version of size: 64 × 64.

CWT is a technique used to represent a signal by convolving wavelets, that vary continuously in transition and scale, with the original signal. The result presents a power spectrum of the signal as in Figure 2. The CWT of a signal x(t) at scale *a* (a>0) and translation *b* is calculated by:

(1) $$X_{w}\left(a,b\right)=\frac{1}{{\left|a\right|}^{1/2}}\int_{-\infty }^{\infty }x\left(t\right)\psi^{*}\left(\frac{t-b}{a}\right)dt$$

where ψ is the mother wavelet which is a continuous function in both the time domain and the frequency domain and the ∗ represents operation of complex conjugate. The mother wavelet is the source that generates daughter wavelets which are the translated and scaled versions of the mother wavelet. After extracting BOLD signals from clusters, the CWT is applied to produce scalograms that provide a detailed representation on these BOLD signals. The scalogram images are then rescaled to 64×64 and fed to multichannel 2D-CNNs for each area. In task-based fMRI experiments, quantifying the change in the BOLD signal across time is significantly important. As mentioned before, CWT scalograms hold information about both frequency and time in an image, and therefore, satisfy this requirement. Applying 2D CNN filters can extract trainable numerical weighted values from these images, during the training phase. During testing phase, these values are compared to classify each entry.

### 3.3. 2D CNN Classification

CNN is a deep learning architecture gaining prominence in the analysis of images, including medical image data. CNN may be characterized by the dimensionality of their convolutional kernels, which in practice is typically between one and four, inclusive. Higher kernel dimensions incur a computational bottleneck, especially when paired with large input sizes, e.g., a 4D CNN that processes fMRI volumetric time series. We have developed a more tractable 2D CNN model four our framework. As a deep neural network, the CNN comprises a number of layers, including convolutional layers based on the aforementioned kernels, pooling layers for reducing the size of the activation map, and fully connected (FC) layers for higher order feature representations.

**Figure 3:**
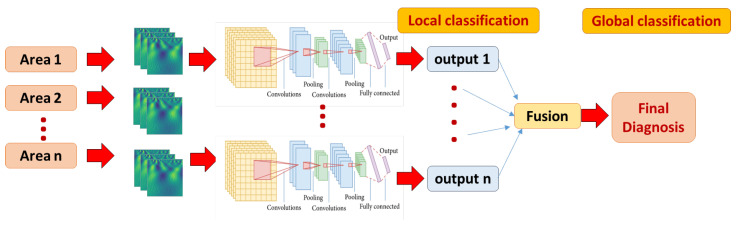
The local and global classification pipeline. A multi-channel 2D CNN local model is developed for each area, fed with corresponding CWT scalograms. The final global classification decision is fused using majority voting approach.

We have extensively tested several model hyper-parameters, as explained in detail in the experimental results. Our CNN model performs three successive passes of convolution and size reduction as shown in Table 1 (which is developed by the model summary method provided by Keras library). These are followed by FC layers (Dense), the final (output) layer having a softmax activation function for purposes of classification. As explained earlier, each brain area is represented with CWT power spectrum images. A separate CNN classifier is developed and tuned for better performance for each brain area. Global classification is obtained with majority voting by all areas, as shown in Figure 3.

## 4. Experimental Results

The incorporated dataset includes 100 subjects (50 ASD and 50 TD). Performance evaluation has been conducted for local CNN model. The whole framework integration is performed using python. The CNN classification model is implemented with Keras library. Several parameters at each step on the proposed spatial dimensionality reduction and classification pipeline are evaluated. The 4-fold average classification accuracy with random shuffling is the score to be optimized. For clustering, 3 clusters provide discriminant average BOLD signals for each area. In the CWT stage, 32, 64 and 128 number of scales have been evaluated. best performance is obtained by 64 scales. Some wavelets have been tested such as: Mexican Hat, Gaussian Derivative and Morlets. Best results are obtained with Morlets.

A grid search method to determine classification parameters has been applied: number of filter (5, 10, 15), CNN kernal sizes (3, 5, 7), epchs (5:70 in order of 5), batch sizes (1, 32, 64, 100) learning rates (0.1, 0.001, 0.0001), optimizers (‘SGD’, ‘Adagrad’, ‘RMSprop’, ‘Adadelta’, ‘Adamax’, ‘Adam’, ‘Nadam’), network activations (‘softplus’, ‘softmax’, ‘softsign’, ‘tanh’, ‘relu’, ‘sigmoid’, ‘linear’, ‘hard_sigmoid’), and finally, kernal weight initializers (‘uniform’, ‘normal’, ‘lecun_uniform’, ‘zero’, ‘glorot_uniform’, ‘glorot_normal’, ‘he_uniform’, ‘he_normal’). The parameters that achieved best results are represented in Table 2. 15 kernels, each with the size of 3 × 3, achieve better results. According to these parameters, the output shape and parameter columns in Table 1 are determined. The number of parameters is the number of trainable network weights at each stage. Only the convolutional and Dense layers contain trainable weights. The maxpooling layers (with size 2 × 2) only calculate the maximum without including a bias parameter. More explanation about how the model layer sizes are determined is provided in [39].

### 4.1. Local Classification

Each local CNN classifier is fed with CWT scalogram images extracted from both hemisphere and the inferior and posterior division, if present. Hence, each classifier has different number of extracted signals for it’s input. Table 3 demonstrates the classification accuracy, sensitivity, specificity, and area under the curve (AUC) for the STG, CG, AG, and PAC areas. The AUC is an effective measure of sensitivity and specificity for assessing inherent validity of the proposed system. Higher AUC means that the proposed system is accurate in differentiating ASD with TD subjects. This implies both sensitivity and specificity are maximum and errors (false positive and false negative) are minimum.

**Figure 4:**
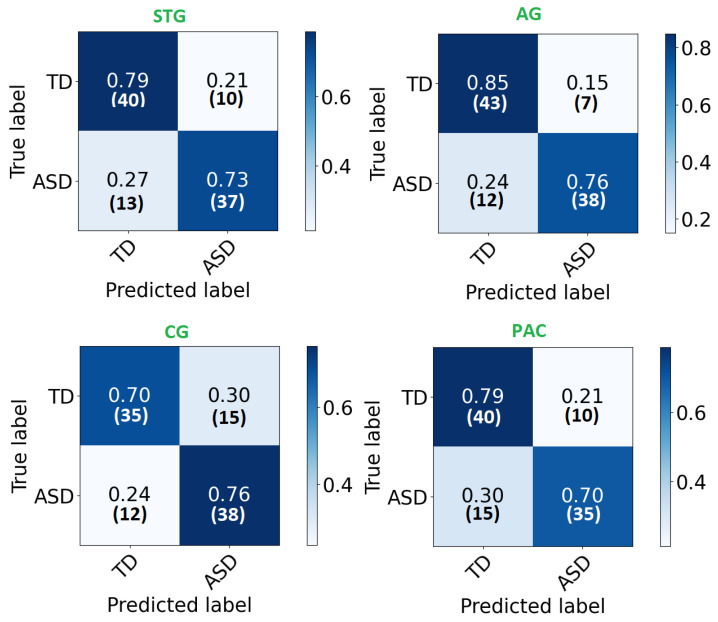
The confusion matrix for each ROI local classifier represented in percentage (number) for each row.

**Figure 5:**
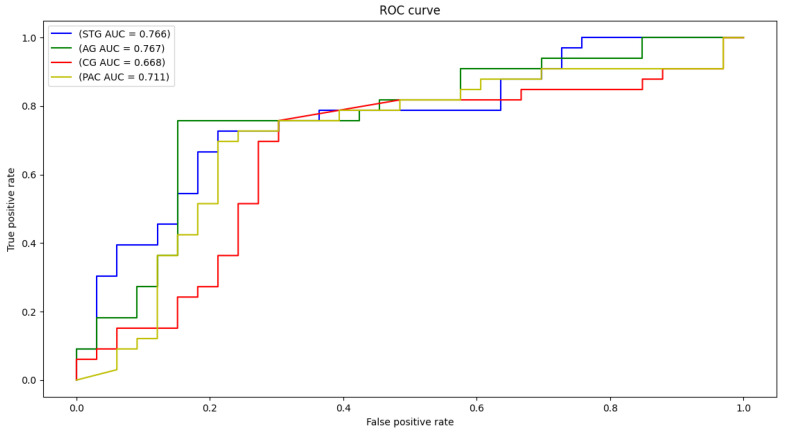
ROC curves and AUC for STG, CG, AG, and PAC selected areas.

The confusion matrix of each area is demonstrated in Figure 4. As can be noted, high percentages are concentrated in the diagonal of each matrix (True positive and True negative) and ranges around the corresponding total accuracy. Therefore, each matrix is balanced. Moreover, receiver operating characteristic (ROC) curves are plotted in Figure 5. After developing local 2D-CNN models, brain maps for each subject are created to represent the level of autism severity for each brain area.

### 4.2. Global Classification

**Figure 6:**
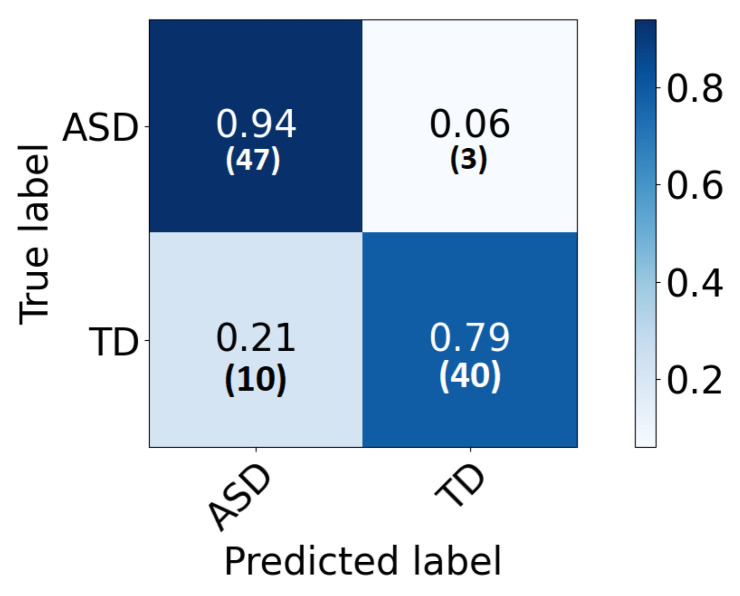
The confusion matrix for the global classifier represented in percentage (number) for each row.

**Figure 7:**
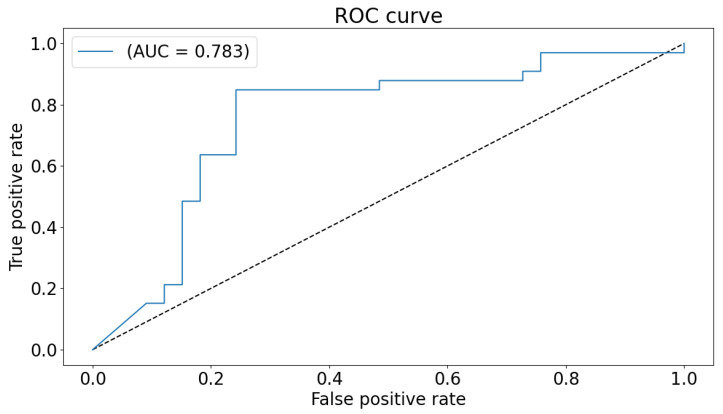
ROC curve and AUC for the global classifier.

The global classification accuracy is obtained by fusing the decision from each local classifier with majority voting. The achieved accuracy is 86% ( sensitivity 82%, specificity 92% ). The confusion matrix is demonstrated in Figure 6. Same notes can be concluded from the confusion matrix. We have also tested a global 2D-CNN classifier that is trained with the scalogram images of all areas at once. This step is performed as a validation step and to highlight the advantage of classification that is based on local classifiers. The obtained accuracy is 82%. Figure 7 plots the ROC of the classifier.

The accuracy is close to the global accuracy of 86% which proves the stability of the system. The inferred reason for less accuracy can be related to the fact that higher number of input features (and hence higher number of parameters) introduced in the CNN network achieves lower accuracy. Therefore in this validation model, the increased number of channels increases the number of parameters and hence, leads to lower performance.

The proposed framework achieves higher accuracies compared to other previous work performed on task-based fMRI scans of the same experiment, as presented in Table 4. A direct comparison between our research and other literature of other tasks would not be objective as other researches incorporate different data sets and task-based fMRI experiments. As a comparison with our previous approaches in [19,20,38], we can note that the accuracy of the proposed classification that is based on local classifiers is higher. The reason is believed to be the better learning of CNN local networks that have lower number of parameters. Majority voting reflects the advantage of building the decision based on the most affected brain areas, rather than all included areas.

### 4.3. Brain Maps

**Figure 8:**
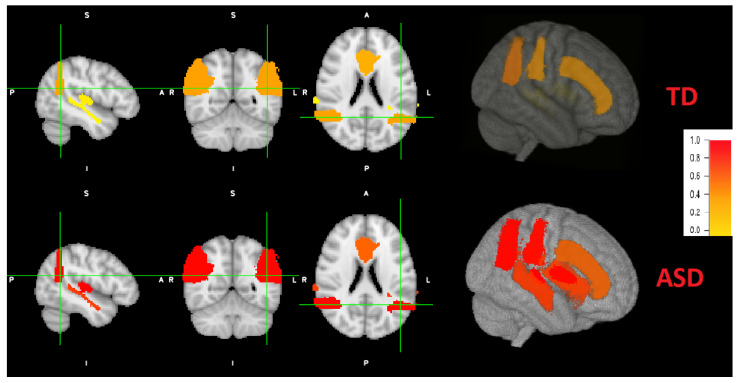
Coronal, sagital and axial 2D views and a 3D view of both ASD and TD example. Brain areas for the ASD individual are more severely distributed (red highlights) than TD peer (more yellow highlight distribution).

According to literature, not every brain area is affected by the same degree for each individual. Therefore, we obtain individual brain maps that explain the level of autism for each area. After the implementation and training of local classifiers, each subject’s local brain area data is tested for each corresponding trained network. The resulted probabilities are represented in a brain map as demonstrated in Figure 8. As an example, the probabilities obtained for the first individual are: (STG: 0.037, AG: 0.36, CG: 0.31, PAC: 0.072). According to majority voting, the four areas has high probabilities for autism (*p* > 0.5), hence, this individual is TD. For the other individual, the obtained probabilities are: (STG: 0.77, AG: 0.97, CG: 0.61, PAC: 0.99). According to majority voting, the four areas has low probabilities for autism (*p* <= 0.5), hence, this individual is TD. Some individuals might have autistic areas and non autistic ones, as mentioned before. An example for the probability distribution (STG: 0.43, AG: 0.8, CG: 0.61, PAC: 0.99). Three areas are autistic (*p* > 0.5) and one area is non autistic (*p* < 0.5). Therefore, this subject is classified as autistic.

Figure 8 also demonstrates a 3D view. The viewing tool is FSLeyes through FSL. As can be noted, the grade of autism are higher (red colors) of ASD subjects, with variable grade on each area. The grade of autism for TD subjects is lower (yellow colors) with different grades.

## 5. Conclusions and Future Work

In this paper, a novel CNN Deep learning based ASD local and global diagnosis system is introduced. The proposed system utilized task-based fMRI to achieve this goal. According to the response to speech experiment, hypoactivation of the bilateral superior temporal gyrus, bilateral primary auditory cortex, cingulate gyrus and angular gyrus are exhibited in ASD toddlers. Whereas, TD peers exhibited typical lateralized activation. Based on these results, local spatial and temporal features are extracted from each ROI separately. CWT is performed to extract scalogram images, from the extracted BOLD signals from spatially reduced clusters, that hold frequency specifications. A local CNN classifier is utilized for each area. Experimental results are reported for all activated brain areas. Accuracies range between 71% and 80%. Global classification is obtained from local results. Achieved accuracy is 86% (with 82% sensitivity and 92% specificity). Finally, local individual brain maps are created for each subject that indicate level of ASD severity.

Future work will include the application of the same approaches on rest-state fMRI of same dataset. Hence, a detailed report for each subject will be obtained for connected brain networks during rest and activated brain areas during task activities. Global decision will be more accurate and will consider all functional aspects of the brain. Researchers are encouraged to collect more data from different geographical sites. A protocol for generic experimental design is recommended to enable researchers to validate their work with other datasets. More validation steps will be performed, leading to a robust ASD diagnosis system. In addition, our future work will include genomic data (which is available in the collected data set used in this paper) to correlate affected brain areas with specific genome sequences to help in early ASD detection. Finally, local classification results will be investigated to identify malfunctioned neuro-circuits involved with ASD.

## Figures and Tables

**Table 1:** CNN network summary.

Layer	Output Shape	Param #
2Dconv	(None, 62, 62, 15)	1635
Max_pooling2D	(None, 31, 31, 15)	0
2Dconv_1	(None, 29, 29, 15)	2040
Max_pooling2D_1	(None, 14, 14, 15)	0
2Dconv_2	(None, 12, 12, 15)	2040
max_pooling2D_2	(None, 6, 6, 15)	0
Flatten	(None, 540)	0
Dense	(None, 10)	5410
Dense_1	(None, 2)	22
Total parameters: 11,147	Trainable parameters: 11,147	

**Table 2:** CNN and CWT parameters.

Kernels	Kernel Size	Learning Rate	Batch Size	Optimizer	Network Activation	Kernel Initializer	Wavelet	Scales	Time Course Normalization
15	3	0.01	32	Adamax	Relu	Lecun_uniform	Morlet	64	Percent signal change

**Table 3:** Accuracy, sensitivity, specificity, AUC of selected ROIs.

Classifier	Accuracy	Sensitivity	Specificity	AUC
STG	0.742	0.74	0.77	0.76
AG	0.80	0.78	0.83	0.77
CG	0.72	0.74	0.71	0.67
PAC	0.71	0.72	0.77	0.71

**Table 4:** A comparison of the proposed CAD system with other GLM-based methods.

Method	Data Source	No. of Subjects	Modeling of BOLD	Classifier	Validation	Accuracy
[38]	NDAR	100 (50 ASD, 50 TD)	DWT	2D CNN	4-fold	78%
[19]	NDAR	60 (30 ASD, 30 TD)	GLM	SNCAE	4-fold	76%
[20]	NDAR	39 (13 Mild, 13 Moderate, 13 Severe)	GLM	RF	10-fold	72%
proposed	NDAR	100 (50 ASD, 50 TD)	CWT	multi-channel 2D CNN	4-fold	86%

## Data Availability

The dataset adopted in this research is provided by the national database for autism research NDAR: http://ndar.nih.gov (accessed on 22 June 2021).

## Supplementary Materials

The following are available online at: https://www.mdpi.com/article/10.3390/s21175822/s1 and https://cisasuedu-my.sharepoint.com/:u:/g/personal/reem_t_haweel_cis_asu_edu_eg/EYfDlDjzwEJDh3Sw_U8Cf7MBiW31QmgDaKpXLnupL77LGA?e=4%3aNDAvwR&at=9.

## References

[1] Amaral D.G., Schumann C.M., Nordahl C.W. (2008). Neuroanatomy of autism. Trends Neurosci..

[2] Gotham K., Pickles A., Lord C. (2012). Trajectories of autism severity in children using standardized ADOS scores. Pediatrics.

[3] Gotham K., Pickles A., Lord C. (2009). Standardizing ADOS scores for a measure of severity in autism spectrum disorders. J. Autism Dev. Disord..

[4] Manning-Courtney P., Murray D., Currans K., Johnson H., Bing N., Kroeger-Geoppinger K., Sorensen R., Bass J., Reinhold J., Johnson A. (2013). Autism spectrum disorders. Curr. Probl. Pediatr. Adolesc. Health Care.

[5] Zwaigenbaum L., Bryson S., Rogers T., Roberts W., Brian J., Szatmari P. (2005). Behavioral manifestations of autism in the first year of life. Int. J. Dev. Neurosci..

[6] Casanova M.F., El-Baz A., Suri J.S. (2017). Autism Imaging and Devices.

[7] Ismail M.M.T. (2016). A CAD System for Early Diagnosis of Autism Using Different Imaging Modalities. Ph.D. Thesis.

[8] Van Horn J.D., Grethe J.S., Kostelec P., Woodward J.B., Aslam J.A., Rus D., Rockmore D., Gazzaniga M.S. (2001). The Functional Magnetic Resonance Imaging Data Center (fMRIDC): The challenges and rewards of large–scale databasing of neuroimaging studies. Philos. Trans. R. Soc. Lond. B Biol. Sci..

[9] Casanova M.F., El-Baz A.S., Suri J.S. (2013). Imaging the Brain in Autism.

[10] Philip R.C., Dauvermann M.R., Whalley H.C., Baynham K., Lawrie S.M., Stanfield A.C. (2012). A systematic review and meta-analysis of the fMRI investigation of autism spectrum disorders. Neurosci. Biobehav. Rev..

[11] Eyler L.T., Pierce K., Courchesne E. (2012). A failure of left temporal cortex to specialize for language is an early emerging and fundamental property of autism. Brain.

[12] Lombardo M.V., Pramparo T., Gazestani V., Warrier V., Bethlehem R.A., Barnes C.C., Lopez L., Lewis N.E., Eyler L., Pierce K. (2018). Large-scale associations between the leukocyte transcriptome and BOLD responses to speech differ in autism early language outcome subtypes. Nat. Neurosci..

[13] Lombardo M.V., Pierce K., Eyler L.T., Barnes C.C., Ahrens-Barbeau C., Solso S., Campbell K., Courchesne E. (2015). Different functional neural substrates for good and poor language outcome in autism. Neuron.

[14] Lindell A.K., Hudry K. (2013). Atypicalities in cortical structure, handedness, and functional lateralization for language in autism spectrum disorders. Neuropsychol. Rev..

[15] Seery A.M., Vogel-Farley V., Tager-Flusberg H., Nelson C.A. (2013). Atypical lateralization of ERP response to native and non-native speech in infants at risk for autism spectrum disorder. Dev. Cogn. Neurosci..

[16] Mody M., Manoach D.S., Guenther F.H., Kenet T., Bruno K.A., McDougle C.J., Stigler K.A. (2013). Speech and language in autism spectrum disorder: A view through the lens of behavior and brain imaging. Neuropsychiatry.

[17] Haweel R., AbdElSabour Seada N., Ghoniemy S., ElBaz A. (2021). A review on autism spectrum disorder diagnosis using task-based functional mri. Int. J. Intell. Comput. Inf. Sci..

[18] Zhuang J., Dvornek N.C., Li X., Yang D., Ventola P., Duncan J.S. Prediction of pivotal response treatment outcome with task fMRI using random forest and variable selection. Proceedings of the 2018 IEEE 15th International Symposium on Biomedical Imaging (ISBI 2018).

[19] Haweel R., Dekhil O., Shalaby A., Mahmoud A., Ghazal M., Khalil A., Ghoniemy S., Keynton R., Elmaghraby A., Barnes G. Functional magnetic resonance imaging based framework for autism diagnosis. Proceedings of the 2019 Fifth International Conference on Advances in Biomedical Engineering (ICABME).

[20] Haweel R., Dekhil O., Shalaby A., Mahmoud A., Ghazal M., Khalil A., Keynton R., Barnes G., El-Baz A. A Novel Framework for Grading Autism Severity Using Task-Based FMRI. Proceedings of the 2020 IEEE 17th International Symposium on Biomedical Imaging (ISBI).

[21] Haweel R., Dekhil O., Shalaby A., Mahmoud A., Ghazal M., Keynton R., Barnes G., El-Baz A. A Machine Learning Approach for Grading Autism Severity Levels Using Task-based Functional MRI. Proceedings of the International Conference on Imaging Systems and Techniques (IST’19).

[22] Haweel R., Shalaby A., Mahmoud A., Ghazal M., Seada N., Ghoniemy S., Casanova M., Barnes G., El-Baz A. (2021). A Novel Grading System for Autism Severity Level Using Task-based Functional MRI: A Response to Speech Study. IEEE Access.

[23] Zhang R., Xu P., Guo L., Zhang Y., Li P., Yao D. (2013). Z-score linear discriminant analysis for EEG based brain-computer interfaces. PLoS ONE.

[24] Bai J., Ding B., Xiao Z., Jiao L., Chen H., Regan A.C. (2021). Hyperspectral Image Classification Based on Deep Attention Graph Convolutional Network. IEEE Trans. Geosci. Remote Sens..

[25] Subasi A., Ercelebi E. (2005). Classification of EEG signals using neural network and logistic regression. Comput. Methods Programs Biomed..

[26] Wen D., Wei Z., Zhou Y., Li G., Zhang X., Han W. (2018). Deep learning methods to process fMRI data and their application in the diagnosis of cognitive impairment: A brief overview and our opinion. Front. Neuroinform..

[27] Hu J., Kuang Y., Liao B., Cao L., Dong S., Li P. (2019). A Multichannel 2D Convolutional Neural Network Model for Task-Evoked fMRI Data Classification. Comput. Intell. Neurosci..

[28] Li X., Dvornek N.C., Papademetris X., Zhuang J., Staib L.H., Ventola P., Duncan J.S. 2-channel convolutional 3D deep neural network (2CC3D) for fMRI analysis: ASD classification and feature learning. Proceedings of the 2018 IEEE 15th International Symposium on Biomedical Imaging (ISBI 2018).

[29] Amin A.E. (2014). Automatic machine fault diagnosis based on wavelet transform and probabilistic neural networks. Int. J. Intell. Comput. Inf. Sci..

[30] Lessa P.S., Sato J.R., Cardoso E.F., Neto C.G., Valadares A.P., Amaro E. (2011). Wavelet correlation between subjects: A time-scale data driven analysis for brain mapping using fMRI. J. Neurosci. Methods.

[31] García J.G.S., López J.M.H., Barbosa E.M., Méndez J.R., Alonso B.d.C. (2016). Diagnosis of ADHD children by wavelet analysis. AIP Conf. Proc..

[32] Chanel G., Pichon S., Conty L., Berthoz S., Chevallier C., Grèzes J. (2016). Classification of autistic individuals and controls using cross-task characterization of fMRI activity. NeuroImage Clin..

[33] Dvornek N.C., Yang D., Ventola P., Duncan J.S. (2018). Learning Generalizable Recurrent Neural Networks from Small Task-fMRI Datasets. Proceedings of the International Conference on Medical Image Computing and Computer-Assisted Intervention.

[34] Westfall J.M., Mold J., Fagnan L. (2007). Practice-based research—“Blue Highways” on the NIH roadmap. JAMA.

[35] Hall D., Huerta M.F., McAuliffe M.J., Farber G.K. (2012). Sharing heterogeneous data: The national database for autism research. Neuroinformatics.

[36] Woolrich M.W., Ripley B.D., Brady M., Smith S.M. (2001). Temporal autocorrelation in univariate linear modeling of FMRI data. Neuroimage.

[37] Jenkinson M., Beckmann C.F., Behrens T.E., Woolrich M.W., Smith S.M. (2012). Fsl. Neuroimage.

[38] Haweel R., Shalaby A., Mahmoud A., Seada N., Ghoniemy S., Ghazal M., Casanova M.F., Barnes G.N., El-Baz A. (2020). A robust DWT–CNN-based CAD system for early diagnosis of autism using task-based fMRI. Med. Phys..

[39] LeCun Y., Bengio Y., Hinton G. (2015). Deep learning. Nature.

